# Rambles in Europe, in 1839

**Published:** 1841-03-27

**Authors:** 


### BIBLIOGRAPHICAL NOTICE.

RAMBLES IN EUROPE, IN 1839. With sketches of *Prominent Surgeons, Physicians, Medical Schools, Hospitals, Literary Personages, Scenery, &c.* By WILLIAM GIBSON, M. D., Professor of Surgery in the University of Pennsylvania, &c. Philadelphia, Lea & Blanchard, 1841.

THIS very agreeable volume is an amplification of the lively sketches of European medical celebrities, which Professor Gibson published in the shape of introductory lectures in the third volume of this journal.

In the style of these lectures, he has added to his account of the distinguished medical men of Paris and London, notices of the most remarkable professional persons whom he encountered in a tour through Great Britain and Ireland, and at the meeting of the Provincial Medical and Surgical Association at Liverpool. Incorporated also with the Rambles, are occasional spirited descriptions of scenery, and other objects of general interest to the tourist, with here and there an allusion to some of the many personages of literary note to whom the author had access. The work is an extremely pleasant one—the style animated and flowing, the personal sketches, of which the volume is chiefly made up, graphic, and to the life. The latter are done in the most kindly spirit, altogether rose-coloured—perhaps, in truth, there is hardly enough of acid in the book to give it a palatable tartness.

As a sample of Dr. Gibson's rare powers as a limner, indeed, as one of the most capital off hand sketches that we have ever read, we extract his notice of Sir Philip Crampton, the great gun of the Dublin profession.

I have spoken, repeatedly, of the leading surgeons and physicians of Dublin, without making allusion to an individual, not to know whom would be "to argue one's self unknown." Need I mention Sir Philip Crampton—better recognized, perhaps, under the expressive appellation of the "*Surgeon-General!*" From all quarters, I had heard of his high reputation and enviable fame, and was not, therefore, surprised, upon reaching Dublin, at the constant exclamation, "You have seen, of course, our Napoleon of surgery." It may well be imagined I should have lost no time in forming the acquaintance of a man, by common consent, thus signalized, even if not attracted by familiarity with his writings. Unqualified praise is too apt, we all know, to lead to disappointment, and, from possibility of such result, I confess I approached Sir Philip's mansion, in Merion Square, with mingled feelings of pleasure and apprehension. Fortunately there was *no* disappointment; for, before reaching the door, I saw a tall, fine-looking man, come out with the apparent intention of springing at once into his cab and of driving off, but who loitered an instant, as I drew near, and seemed, by an intuitive glance, to understand I was seeking him. I had scarcely time to tell my name before he directed the servant, by a sign, to put the carriage away, and the next moment, with gracious pressure of the hand, led me into the house—where I remained two hours, buried among books and instruments, reclining upon old-fashioned chairs and sofas of the most comfortable and luxurious kind, or pacing the room, listening to one continued strain of the most lively and agreeable declamation on all subjects, but, chiefly, surgical; examining curiously-contrived and ingenious instruments; answering questions in relation to the Marchioness W . . . . . . y—whom he had known and attended as the Lady of the Lord Lieutenant—and other distinguished Americans; with replies and anecdotes of some of the characters mentioned, strikingly illustrative of their peculiarities, strong points, and appearance, dwelling upon favourable traits and cautiously avoiding, in every instance, censure and illiberality. In particular I was much amused at the account of his first meeting with Washington Irving. Being the intimate friend of Tom Moore, he had called, whilst on a visit to London, at his lodgings, to see him, and was told at the door, by an old female domestic, that Mr. Moore was at home, but would see no one except Mr. Washington Irving, when Sir Philip instantly exclaimed, "Oh, I'm Washington Irving," and immediately rushed by, hotly pursued by the old woman, who seized the tail of his coat and loudly cried out, "No, sir, no, sir, you are not Mr. Irving, come down, sir, come down, sir, you can't go up stairs," while Sir Philip, making desperate efforts to escape, and fearful of his coat being torn, replied, "let me go, let me go, my good woman, I tell you I *am* Washington Irving," when, in the midst of all the hubbub, who but the real Simon Pure should appear—hearing his name repeatedly vociferated—at the head of the stairs, exclaiming, at the same time, "*You* are Mr. Washington Irving, are you, the deuce you are, and so am I." "Never," said Sir Philip, "in my life did I feel half so mortified, being completely caught in my own trap, for Irving had been sitting, for some time, in Moore's room, talking to him as he lay in bed, and nothing but my friend's recognition of my voice relieved my embarrassment, by his crying out, "Come in Crampton, come in, I'll introduce you to Irving."

Several times I rose to take leave of Sir Philip, thinking I should interfere with professional calls; but he always said, "Sit still, sit still, I'll tell you when I *must* go; for I don't always get a chance of chatting with a foreign surgeon; and yet, after all, I don't know that I ought to call you a foreigner, when your people have so much English, Irish, and Scotch blood in their veins, and so much of our own cleverness about them." After a while he rose and said, "Come, I never knew any American but was fond of horses and dogs, go out and look at mine." Accordingly his man John—an active, sprightly little Irish groom, who, from the stir and bustle he made, and respectful attention to every gesture of his master, looked as if he would have, willingly, broken his neck for him, any moment—was summoned, and with the quickness of lightning flung open the stable doors, exhibiting, one by one, some of the finest cobs, colts, and bay geldings, for saddle and harness, I ever beheld, always reserving the best for the last, and, knowingly, asking my opinion of each, merely for the opportunity of showing his own skill, should I chance to be at fault—in which sport, for love of the science of horsemanship, Sir Philip, occasionally, joined. The animals, however, were all so much alike, in quality, being very high-bred, and possessed, in an eminent degree, of so many points valued by horsemen, as to render it difficult for a more experienced judge than myself to discover their peculiarities and respective traits, notwithstanding they were put through various gaits, and their action, skilfully displayed, in an open space near the stable, which seemed contrived, purposely, for such exhibitions. After having, carefully, examined the whole, however, I said, I think, Sir Philip, if allowed to choose, out of the six, fine as they are, I should select for my own use that chestnut-sorrel mare, pointing out, at the same time, a splendid creature, about fifteen hands and an inch high, with neck like a rainbow, sharp, peaked, squirrel-like ears, small, delicate head and muzzle, and dished face, with red, expanded nostrils, short back, close coupling, and barrel, stifle, legs, and whole finish, coming as near perfection, as any thing I could well imagine. "Ah, said he, "you really have pitched upon the best nag in the stable, though she is twenty years old, for often she has carried my weight, which you see is no trifle, over the highest walls and hedges about Dublin, where many a larger animal would have broken my neck and its own too." "Well," said he, "I see you *are* a horseman, now look at my dogs, and let me know if I can say of you 'Equis canibusque gaudet;' here Dash, here you villain, show yourself. John, look at the bull-terrier under that colt's heels; I would not have him hurt for fifty guineas; not such a pup in Dublin, sir. Now tell me what you think of Dash; have you such a setter in your country? Afraid of nothing, no brier can touch him, staunch as a rock. There, again, look at that black slut; is she not a beauty? should like to see her match. But before we go to the house, you *must* feel the weight of that bull pup, not heavier, you see, than a large rat, and as active as a weasel; I never saw one before with white ground, so dotted with such peculiar dark spots. Now, John, bring round the cab; sorry I *must* leave you, my dear sir, but the *Lord Lieutenant* is ill, and will be expecting me by this time. Come and dine with me to-morrow, and I'll give you grouse and venison; precisely at seven, if you please. But stop, just step into the back parlour, one moment, and look at the pictures of my horses. Ah, that which has caught your eye, was the best nag I ever owned; but Lord ——— *would* have him, and I could not refuse, and though it went hard with me to part with him, I let him go at cost—a hundred and fifty pounds—when he would have brought, in the market, three hundred. And now, good-bye again, and remember the grouse at seven to-morrow."

At the appointed hour I rode to Merion Square, and was introduced by Sir Philip, to his accomplished daughters, and regretted not seeing his sons, some of whom were abroad, holding, I believe, diplomatic stations. His nephew, Mr. John Hamilton, a young surgeon of great promise, whom I had previously seen much of, I then also had the pleasure to meet. Sir Philip apologized for not inviting other company, saying, he wished to have me all to himself, and to inquire a great deal about American surgeons and physicians, of whom he had heard so much, and to show me some lithotriptic and other instruments, I had not seen at my first visit. Accordingly, after partaking, plentifully, of a fine salmon with its savoury shrimp sauce, southdown mutton, grouse, just received from Scotland, mountain venison and plumb pudding, all served in courses; and having regaled ourselves with the best bottle of claret—for which Dublin has always been famous—I had tasted in Europe, we had full leizure to discuss, till a late hour of the night, many a topic it would be impossible to introduce here, but which enabled me to form a better estimate of Sir Phillip's powers, varied professional and general information, than I could have obtained, perhaps, in a month, from ordinary or casual intercourse. From this, and previous, and subsequent opportunities, then, I arrived at the conclusion, that few men in any country—from very long experience and extensive practice in a large community, from extraordinary natural endowments, uncommon tact and observation, extreme caution and prudence, aided by the best intellectual education, profound research, and professional reading, combined with powers of intense application, the result of quickness of perception and enthusiasm, and long and intimate association with persons of the highest rank and literary reputation—can be found superior to Sir Philip Crampton. Independently, however,of native intellectual and acquired professional advantages, there can be no doubt that Sir Phillip's great success and reputation may be attributed, in some measure, to personal attraction, and to refined and elegant manners ; for of all the individuals it has been my lot to meet in European or American society, no one, in such endowments and accomplishments, has approached so near the standard of excellence or perfection.* It may easily be conceived, indeed, that a man full six feet high, strait as an arrow, in frame and muscle a Hercules, in grace and dignity an Apollo, with head beautifully moulded, and features regular, animated, and irresistibly attractive, "an eye like Mars to threaten and command," with address and blandishments so winning, fascinating, and seductive, as to excite the admiration of every beholder, must, when possessed of the higher attributes enumerated, be well calculated to produce a powerful impression, and to exercise unlimited influence even over the strongest minds. So great, indeed, are said to be his tact and knowledge of human nature, as to enable him to read, at a glance, individual character, and adapt, without effort, his conversation and manners to the person addressing him.

Sir Philip Crampton has long been connected, more or less, with the best Dublin hospitals, as a lecturer and operative surgeon, though not attached now, I believe, to any of them—except as a consulting surgeon. As a writer, too, he has been most advantageously known almost from the commencement of his career. His first essay was a valuable one on "*Entropeon*;" and subsequently, numerous papers, or lectures, generally on difficult or obscure practical points in surgery, have appeared in the different British medical journals, all characterised by great force and originality, and most happily illustrated by apposite facts and observations. The chief of these are, on dislocations of the shoulder and other joints, on fractures, on burns and scalds on the qualifications of a surgeon, and are contained in the Dublin Journal of Medical and Chemical Science. An excellent essay also appeared, separately, in 1839, entitled An Outline of the History of Medicine; and still more recently, a very valuable and original lecture on the "*Chamelion*," in which some important discoveries are detailed, in relation to the habits of that extraordinary animal. Of most of these, however, I shall speak in another publication.

Of Sir Charles Bell, the author's old preceptor, he gives a glowing sketch. Sir Charles distinguished as he is, as a writer and lecturer, it seems has a small share of private practice, which Dr. Gibson thus accounts for in a strain of comment, intended for general application.

How does it happen, then, he has not enjoyed the extensive practice, and become so enormously rich as some of the European surgeons we hear of? This is easily understood, when recollected, that the facility of gaining practice does not, invariably, depend upon amount of intellect or extent of individual qualification; that many persons, of very limited capacity and meager acquirements, possess, inherently, the faculty of pleasing, and even fascinating the public—who are no judges of professional merit—to a great degree; that others cultivate, as a business, the means of obtaining professional livelihood, independently of professional knowledge, and resort to every stratagem and device which self-preservation suggests, in order to place them on a level, or force themselves above their intellectual rivals. So that many a man, with only the manners of a waiter and the intellect of a mouse, has obtained enormous business, whilst his talented brother has actually starved, or, perhaps, been thrown into prison. Such is well known to be the case, not only in our profession, but in law and divinity, and more or less, perhaps, in every other vocation, not only in Europe but in this country, and, indeed, throughout the world. Now Sir Charles, from early life, has been employed, most laboriously, in studying and teaching his profession; in writing and publishing large and valuable works; in hospital practice; in making collections of human and morbid anatomy, and pictures for class demonstrations, so expensive, as to absorb most of his professional receipts, and leave little time for playing the fine gentleman, for bowing, and scraping, and flattering old maids, and young maids, and old women, and noblemen, and gentry; and withal, has been too scrupulously honest, upright, and independent in sentiment, to stoop to any measures, however well calculated to advance his fortune, between which and strict professional dignity and honour he found vast incompatibility. I do not mean to infer, how-

*Among the *on dits* of Dublin, respecting Crampton's figure, though I do not vouch for its authenticity, is one strikingly characteristic—that Sir Thomas Lawrence, struck with the perfect formation of his lower extremities, and being engaged in his full-length portrait of John Kemble, requested Sir Philip, then on a visit to London, to stand for *Kemble's legs*.

ever, from what I have just said, that all who obtain extensive practice must, necessarily, resort to unworthy means. The reverse I know to be the case, in many instances; nor do I wish to be understood to say, that Sir Charles had not succeeded, at all, in obtaining business; but only to imply, that his private professional calls, though numerous and important, were much fewer than if he had laid himself out for practice, by cultivating the arts of obtaining and securing it. This much, however, is certain, that any man of moderate capacity, who, for years, has been a hospital surgeon, will see more, in two or three months, than any one, engaged in private practice only, in a year; and will often reap more, from close investigation of a single case, than another from fifty; so that, after all, it is not the number of cases a medical man may have attended, but the use made of them, which will establish for him the character of a bad or good practitioner.

We conclude with the following humourous sketch of Dr. Graves.

I had heard nothing, it so happened, of the appearance or manners of Dr. Graves, and, therefore, when ushered into his presence in his own parlour, was startled at finding myself looking up to the face of a very tall, slender, handsome, and well-dressed man, whom, if I had met by chance in the street, I should have turned round to look at and inquired of some passer-by who he was. But, before I had time for a second glance, he had turned to one side in the act of presenting me to one of the most beautiful women I had seen in Ireland, who, from her tall, splendid figure, lovely features, dazzling row of pearly teeth, youthful appearance, and graceful movements as she swept across the room, I should have taken for his sister, had he not, simultaneously, announced Mrs. Graves—such was the correspondence, seemingly, between them in person, manners, and address. First impressions are said to be, generally, the best, and I at once settled in my own mind that Graves and I would soon be upon intimate terms, for I quickly perceived a certain indescribable something in his keen, penetrating eye, arrangement of the muscles of the face, and mobility of the tip of his long and delicately-formed aquiline nose, plainly indicative of humour, high spirits, and quizzical propensities, with ample power to subdue or bring them forward at pleasure. And I was not deceived; for, of the many little stories I picked up in the land of blunders and bulls, some of his were the best; told so oddly and quaintly, with the true Tipperary accent on his tongue, when he chose to place it there, that I can't, to this day, help laughing when I think of the queer questions put to some of his hospital patients and the funny dialogues carried on between them, such as, "Patrick, my dear, and how are you to day? Oh bravely, yer honour, and how's yer honour's self? Did the pills throuble you, last night, my dear? Faix, and they handled me nately. Where is your tongue, my dear? In my mouth, yer honour. And are ye maning to bring it out? Troth, and I am. Can you breathe, my lad! Av coorse, yer honour, and sure you might see that I can. That's right, my dear, and there's a pinch of snuff for you, Pat. Thank you kindly, and long life to yer honour; troth and yer a rale gentleman of a doctor." And so he proceeded from bed to bed, and no language can convey the odd mixture of kindness, and humour, and familiarity, and good sense, and soberness, and dexterity with which he managed to draw from the patients the history of their disease, the symptoms, &c. All this and a great deal more was enacted in the *Meath Hospital*, where I spent a forenoon with him, after partaking, at his house, of a plentiful and most luxurious breakfast, such as would have done honour to the best Virginia housewife, or to the table Mrs. Randolph herself. After this I saw as much of Dr. Graves as a fortnight in Dublin would allow. That is, I saw him every day, either at his own house, or at my lodgings, or the Meath Hospital, where he may be found, every morning, peeping and prying into every hole and corner of the building, cracking jokes with the patients or pupils, or old women, or poring over, intently, some medical production, or volume of natural history, or book of travels; for he is very fond of such studies, and took great delight in asking all sorts of questions about our Indians and lakes, and trees and prairies, and cataracts and great rivers, and buffaloes. And, from all I did see of him, I felt justified, I thought, in jumping to the conclusion, that he is a man of very extraordinary abilities, sufficient to enable him to master any subject to which he may devote his attention, and to sift, thoroughly, any medical case and follow out, successively, its treatment; but that he is seldom capable, for any length of time, of devoting himself, soul and body, to any given point, or subject; that he is too fond of analogy and of drawing conclusions from solitary facts, so that his listener is always left in doubt as to the certainty of his deductions, however striking and brilliant they may be, as they generally are, for wanting full confidence in his premises.

